# Barriers to oral care: a cross-sectional analysis of the Canadian longitudinal study on aging (CLSA)

**DOI:** 10.1186/s12903-023-02967-3

**Published:** 2023-05-15

**Authors:** Vanessa De Rubeis, Ying Jiang, Margaret de Groh, Lisette Dufour, Annie Bronsard, Howard Morrison, Carol W. Bassim

**Affiliations:** 1 Department of Health Research Methods, Evidence, and Impact, McMaster University, Hamilton, ON L8S 4L8 Canada; 2 Applied Research Division, Centre for Surveillance and Applied Research, Public Health Agency of Canada, Ottawa, ON K0A 0K9 Canada; 3 Office of the Chief Dental Officer, Public Health Agency of Canada, Ottawa, ON K0A 0K9 Canada; 4 Department of Medicine, McMaster University, Hamilton, ON L8S 4L8 Canada

**Keywords:** Oral health, Barriers, Dental insurance, CLSA

## Abstract

**Background:**

Oral health plays a role in overall health, indicating the need to identify barriers to accessing oral care. The objective of this study was to identify barriers to accessing oral health care and examine the association between socioeconomic, psychosocial, and physical measures with access to oral health care among older Canadians.

**Methods:**

A cross-sectional study was conducted using data from the Canadian Longitudinal Study on Aging (CLSA) follow-up 1 survey to analyze dental insurance and last oral health care visit. Logistic regression was used to estimate odds ratios (ORs) and 95% confidence intervals (CIs) for the association between socioeconomic, psychosocial, and physical measures with access to oral care, measured by dental insurance and last oral health visit.

**Results:**

Among the 44,011 adults included in the study, 40% reported not having dental insurance while 15% had not visited an oral health professional in the previous 12 months. Several factors were identified as barriers to accessing oral health care including, no dental insurance, low household income, rural residence, and having no natural teeth. People with an annual income of <$50,000 were four times more likely to not have dental insurance (adjusted OR: 4.09; 95% CI: 3.80–4.39) and three times more likely to report not visiting an oral health professional in the previous 12 months (adjusted OR: 3.07; 95% CI: 2.74–3.44) compared to those with annual income greater than $100,000.

**Conclusions:**

Identifying barriers to oral health care is important when developing public health strategies to improve access, however, further research is needed to identify the mechanisms as to why these barriers exist.

**Supplementary Information:**

The online version contains supplementary material available at 10.1186/s12903-023-02967-3.

## Introduction

Maintaining good oral health is an integral aspect of overall health. Adverse oral health outcomes increase with age, and include complete loss of teeth (edentulism); the need to have dental prostheses or false teeth; dental-related problems with chewing and eating; and dry mouth and mouth sores [1, 2]. Poor oral health has been associated with age-related chronic conditions, cognitive impairment, and even premature mortality [3–5] and has been linked to the development of chronic cardiac, pulmonary and metabolic diseases, including diabetes [6–9].

Access to regular oral health care is crucial for the early identification and prevention of oral diseases. Many barriers to accessing care continue to exist, including the cost of dental treatments, lack of transportation, psychosocial factors, including anxiety or depression, and accessibility and availability of oral health providers [10–13]. Additionally, the perception of oral health status is an important factor in oral care utilization [14]. Gaszynska et al. (2014) reported that study participants were over-optimistic about their oral health condition and dental needs, highlighting the disparity between perceived and actual oral health [15]. Similarly, it has been reported that the majority of dentate (with natural teeth) and edentulous (without natural teeth) elderly believe they would not seek oral care until they feel pain, have a chewing problem, or experience social embarrassment [16]. Another study examining edentate individuals reported that 48% of those aged 65–74 and 63% of those aged 75 and older had not accessed oral care for over 10 years [17]. Access to dental insurance is also a barrier that exists and may influence oral care [18]. For instance, in Canada, most oral care is paid for out-of-pocket or through private insurance with select groups receiving insurance through the federal government, which may influence if people regularly see their oral health professional [18, 19]. Disparities in oral health care increase the risk of oral diseases [20, 21]. By examining barriers to oral care and the characteristics of those facing specific barriers, guidelines and solutions may be developed to improve regular and timely oral health care. Previous studies have examined barriers to oral health care; however, differences across jurisdictions and countries have been noted, and cross-sectional studies with small sample sizes have been used, signifying the importance of further exploring barriers using population-based samples [22, 23]. The objectives of this study were to identify barriers to oral care by examining the association between socioeconomic, psychosocial and physical measures with access to oral care (measured by dental insurance and last oral health visit).

## Methods

### Data source and study design

**Fig. 1:**
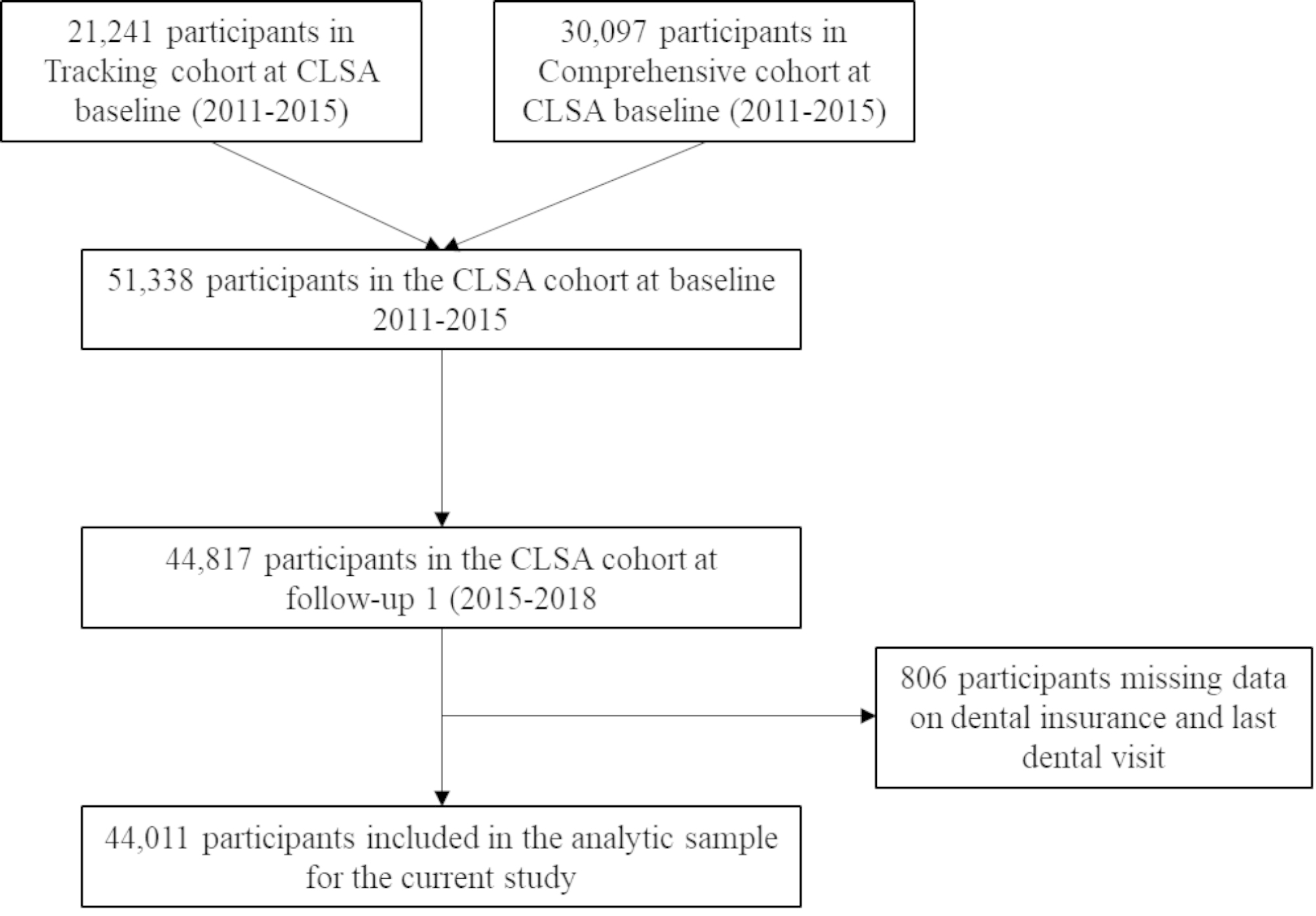
Participant flow diagram describing analytic sample for current study using participants from the Canadian Longitudinal Study on Aging (CLSA)

A cross-sectional study was conducted using data from the Canadian Longitudinal Study on Aging (CLSA). The CLSA is a longitudinal study of Canadians who were between the ages of 45 and 85 at time of recruitment (2011–2015). People were eligible for inclusion if they spoke English or French, were community-dwelling from one of the 10 provinces, did not live on a First Nations reserve or were not a member of the Canadian Armed Forces. Data are planned to be collected every three years for an anticipated 20 years, or until a participant dies or is lost to follow up. At CLSA baseline, 2011–2015, there were 51,338 participants, at follow-up 1, 2015–2018, 44,817 participants were included. A description of the analytic sample for the current study can be found in Fig. 1. A more in-depth description of the CLSA has previously been published [24]. The CLSA is comparable to similar population-based Canadian samples, however, is not representative of the Canadian population [24]. Ethics approval was granted for this study through the Health Canada and the Public Health Agency of Canada Research Ethics Board (REB) (REB project #2014-0015). For the CLSA, ethics approval was granted from each data collection site across Canada [24].

### Oral care outcomes

Oral health care was measured using self-reported dental insurance and oral health visit question, which were taken from CLSA follow-up 1, which was collected from 2015 to 2018.

*Dental insurance.* To measure if participants had dental insurance they were asked, *“What type of dental insurance do you currently have?”* Response options were: (1) private; (2) public; (3) none. This was dichotomized into yes (private or public) versus no.

*Last oral health visit.* Participants were also asked “*When was the last time you visited a dental professional?*”, which was dichotomized into ≤ 12 months versus > 12 months. Participants were also asked about reasons for not visiting an oral health professional. Response options included: (1) not needed (perceived); (2) difficulty getting an appointment; (3) no dentist in the area; (4) no dental hygienists/denturist in the area; (5) transportation problems; (6) personal and family responsibilities; (7) unable to leave house due to health condition. Participants could only select one main reason for not visiting an oral health professional. In addition, participants were specifically asked about cost as a barrier: “*In the past 12 months, have you not gone to a dental professional because of the cost of care?”* Responses included: (1) yes; (2) no.

### Socioeconomic, psychosocial, and physical measures

Socioeconomic, psychosocial, and physical measures were taken from both CLSA baseline (2011–2015) and CLSA follow-up 1 (2015–2018). From CLSA baseline, participant sex (male, female) and education (post-secondary, no post-secondary) were taken. All remaining variables were taken from follow-up 1, including age group (45–54, 55–64, 65–74, 75+); edentulous (yes, no); total household income (<$50,000, $50–100,000, >$100 000); urban/rural status (urban, rural); past 30-day smoking status (daily [≥ 30 cigarettes; i.e., at least one per day], occasional [1–29 cigarettes], none [0 cigarettes]); number of chronic conditions (0, 1 or more). Social support was ascertained using the functional support Medical Outcomes Study (MOS) score, dichotomized into two groups: low social support (bottom 20%) and high social support (above 20% cut-off) [25]. Anxiety and depression were measured by asking participants, “*Has a doctor ever told you that you have an anxiety disorder such as a phobia, obsessive-compulsive disorder or a panic disorder*?” and “*Has a doctor ever told you that you have a mood disorder such as depression (including manic depression), bipolar disorder, mania, or dysthymia*?”, respectively. Both were dichotomized as yes versus no. Similarly, memory problem was measured by asking participants, “*Has a doctor ever told you that you have a memory problem*?” and “*Has a doctor ever told you that you have dementia or Alzheimer’s disease?”.* A yes response to either question was classified as yes, if no was responded to both, it was classified as no.

### Statistical analysis

For all analyses, sampling weights were used. For descriptive statistics such as frequency and percentages, inflation weights were used, and for regression analyses, analytic weights were used. Multivariable logistic regression models were used to estimate odds ratios (ORs) and 95% confidence intervals (CIs), for the association between socioeconomic, psychosocial, and physical measures with (1) dental insurance and (2) last oral health visit. Models were adjusted for all socioeconomic, psychosocial, and physical variables (age group, sex, household income, education, residence, smoking status, mood disorder, anxiety, memory problem, chronic conditions, social support, edentulous, last dental visit). Statistical analyses were conducted using SAS [26]. Complete case analysis was conducted given minimal data was missing.

## Results

### Sample characteristics

After removing those who were missing dental insurance and last dental visit (n = 806), 44,011 adults were included in the study. A complete description of the sample can be found in Table 1. Just over half (51.7%) of the sample were females and were aged 46 to 64 years of age (57.2%) at the time of data collection. Almost 40% of the sample reported not having dental insurance and 15% reported their last dental visit was over 12 months prior to completing the survey.

**Table 1:** Characteristics of Canadian adults from the Canadian Longitudinal Study on Aging (CLSA)

Characteristics	Number of participants (n = 44,011)	Weighted %^1^
Age group		
46–54 55–64 65–74 75–92	6493 14,564 13,112 9842	19.4% 37.8% 26.3% 16.5%
Sex		
Female Male	22,487 21,524	51.7% 48.3%
Household Income		
<$50,000 $50,000-$100,000 >$100,0000 Missing	11,917 15,025 14,403 2666	25.9% 36.6% 37.5% 6.0%
Education		
Less than post-secondary Post-secondary Missing	10,484 33,426 101	25.4% 74.6% 0.2%
Residence		
Rural Urban Missing	4649 37,384 1978	14.7% 85.3% 4.5%
Smoking Status		
None Occasional Daily Missing	40,787 659 2535 30	92.0% 1.8% 6.2% 0.7%
Mood Disorder		
No Yes Missing	36,016 7525 470	83.4% 16.6% 1.1%
Anxiety		
No Yes Missing	39,554 3994 463	90.9% 9.1% 1.1%
Memory problem or dementia/Alzheimer’s		
No Yes Missing	42,680 875 456	98.0% 2.0% 1.0%
Chronic conditions		
No One or more Missing	3652 40,127 232	10.3% 89.7% 0.5%
Social support		
No Yes Missing	8428 34,237 1346	16.9% 83.1% 3.1%
Edentulous		
No Yes Missing	40,845 3162 4	92.6% 7.3% 0.01%
Dental Insurance		
No Yes Missing	16,891 26,903 217	39.8% 60.2% 0.5%
Last Dental Visit		
≤12 months >12 months Missing	37,439 6512 60	85.1% 14.9% 0.1%

1. Parentage missing not included in total percent

### Dental insurance

The prevalence of dental insurance by socioeconomic, psychosocial, and physical measures can be found in Table 2. People with a household income of less than $50,000, compared to those with a household income greater than >$100,000 (adjusted OR: 4.09; 95% CI: 3.80–4.39), resided in a rural residence, compared to urban (adjusted OR: 1.36; 95% CI: 1.26–1.48), reported no natural teeth (edentulous), compared to those who had at least one natural tooth (adjusted OR: 1.39; 95% CI: 1.15–1.44) and reported their last dental visit greater than 12 months, compared to those who visited an oral health professional within the past 12 months (adjusted OR: 2.62; 95% CI: 2.42–2.83) had greater odds of not having dental insurance. Whereas people aged 46–54 years had greater odds of having dental insurance compared to all other age groups (Table 2).

**Table 2:** Adjusted association between various characteristics and not having dental insurance among Canadian adults from the Canadian Longitudinal Study on Aging (CLSA) at CLSA follow-up 1 (2015–2018)

Characteristics	Dental Insurance (Weighted %)		Adjusted OR^1^ (95% CI)
	Insurance	No Insurance	
Age group			
46–54 55–64 65–74 75–92	77.1% 69.1% 47.1% 40.6%	22.9% 30.9% 52.9% 59.4%	1.00 1.35 (1.25–1.47) 2.59 (2.38–2.82) 2.94 (2.68–3.22)
Sex			
Female Male	57.7% 62.9%	42.3% 37.1%	1.10 (1.05–1.16) 1.00
Household Income			
<$50,000 $50,000-$100,000 >$100,0000	35.5% 60.6% 78.3%	64.5% 39.4% 21.7%	4.09 (3.80–4.39) 1.79 (1.68–1.91) 1.00
Education			
Less than post-secondary Post-secondary	51.6% 63.2%	48.4% 36.8%	1.04 (0.98–1.10) 1.00
Residence			
Rural Urban	50.4% 61.9%	49.6% 38.1%	1.36 (1.26–1.48) 1.00
Smoking Status			
None Occasional Daily	60.6% 60.4% 54.2%	39.4% 39.6% 45.2%	1.00 1.21 (0.97–1.51) 1.06 (0.95–1.19)
Mood Disorder			
No Yes	59.9% 61.6%	40.1% 38.4%	1.00 0.95 (0.87–1.02)
Anxiety			
No Yes	60.1% 61.8%	39.9% 38.2%	1.00 0.96 (0.87–1.05)
Memory problem or dementia/Alzheimer’s			
No Yes	60.3% 56.8%	39.7% 43.2%	1.00 0.87 (0.73–1.03)
Chronic conditions			
No One or more	66.7% 59.4%	33.3% 40.6%	1.00 0.94 (0.85–1.03)
Social Support			
No Yes	54.9% 61.9%	45.1% 38.1%	1.00 (0.94–1.07) 1.00
Edentulous			
No Yes	62.6% 30.1%	37.5% 69.9%	1.00 1.39 (1.15–1.44)
Last Dental Visit			
≤12 months >12 months	64.8% 34.1%	35.2% 65.9%	1.00 2.62 (2.42–2.83)

1. Adjusted for all covariates in the table (age group, sex, household income, education, residence, smoking status, mood disorder, anxiety, memory problem or dementia/Alzheimer’s, chronic condition, social support, edentulous and last dental visit)

### Last dental visit

The characteristics of those who visited a dentist within the last 12 months, and those who did not are in Table 3. People who reported no natural teeth were almost 10 times (adjusted OR: 9.95; 95% CI: 8.90-11.13) more likely to report not seeing an oral health professional within the past 12 months, compared to those who reported having at least one natural tooth. People with an income less than $50,000 (adjusted OR: 3.07; 95% CI: 2.74–3.44) and a household income from $50,000-$100,000 (adjusted OR: 1.77; 95% CI: 1.60–1.97) compared to those with an income greater than $100,000, had greater odds of reporting not visiting an oral health professional within the past 12 months. Additionally, those who were daily smokers, compared to those who were not smokers (adjusted OR: 1.84; 95% CI: 1.61–2.11), had no social support, compared to social support (adjusted OR: 1.14; 95% CI: 1.05–1.25), and reported no dental insurance, compared to having dental insurance (adjusted OR: 2.61; 95% CI: 2.41–2.83) were more likely to report they did not visit an oral health professional in the last 12 months.

**Table 3:** Adjusted association between various characteristics and last dental visit > 12 months among Canadian adults from the Canadian Longitudinal Study on Aging (CLSA) at CLSA follow-up 1 (2015–2018)

Characteristics	Last Dental Visit (Weighted %)		Adjusted OR^1^ (95% CI)
	≤ 12 months	> 12 months	
Age group			
46–54 55–64 65–74 75–92	88.1% 87.6% 83.7% 78.0%	11.9% 12.4% 16.3% 22.0%	1.00 0.75 (0.67–0.85) 0.55 (0.49–0.62) 0.56 (0.49–0.64)
Sex			
Female Male	86.1% 84.0%	13.9% 16.0%	0.63 (0.58–0.68) 1.00
Household Income			
<$50,000 $50,000-$100,000 >$100,0000	71.1% 86.5% 93.6%	28.9% 13.5% 6.4%	3.07 (2.74–3.44) 1.77 (1.60–1.97) 1.00
Education			
Less than post-secondary Post-secondary	76.3% 88.1%	23.7% 11.9%	1.53 (1.41–1.65) 1.00
Residence			
Rural Urban	80.5% 86.2%	19.5% 13.8%	1.09 (0.98–1.21) 1.00
Smoking Status			
None Occasional Daily	86.2% 85.2% 67.9%	13.8% 14.8% 32.1%	1.00 1.28 (0.96–1.70) 1.84 (1.61–2.11)
Mood Disorder			
No Yes	85.2% 84.7%	14.8% 15.3%	1.00 0.97 (0.88–1.08)
Anxiety			
No Yes	85.3% 83.0%	14.7% 17.0%	1.00 1.08 (0.95–1.23)
Memory problem or dementia/Alzheimer’s			
No Yes	85.2% 78.3%	14.8% 21.7%	1.00 1.07 (0.85–1.36)
Chronic conditions			
No One or more	87.3% 84.8%	12.7% 15.2%	1.00 0.99 (0.86–1.14)
Social Support			
No Yes	79.8% 86.5%	20.2% 13.5%	1.14 (1.04–1.25) 1.00
Edentulous			
No Yes	89.0% 35.1%	11.0% 64.9%	1.00 9.95 (8.90-11.13)
Dental Insurance			
No Yes	75.3% 91.6%	24.7% 8.4%	2.61 (2.41–2.83) 1.00

1. Adjusted for all covariates in the table (age group, sex, household income, education, residence, smoking status, mood disorder, anxiety, memory problem or dementia/Alzheimer’s, chronic condition, social support, edentulous and dental insurance)

### Reasons for no dental visit in the past 12 months

Reasons for not visiting an oral health professional in the last 12 months are shown in Table 4. Among the 2,562 who reported a reason for not visiting an oral health professional in the past 12 months, the most commonly reported reason was that it was not needed (80.4%), followed by personal and family responsibilities (10.6%). When stratifying reasons for not visiting an oral health professional by insurance, reasons were somewhat similar. However, people without insurance had a slightly higher proportion of people who reported that they did not need to visit an oral health professional; whereas people with dental insurance had a slightly higher proportion of people who reported personal and family responsibilities and difficulty getting an appointment as the reason. Given the large proportion of people who reported a reason for not visiting an oral health professional (n = 2,562; 39.3%), a sensitivity analysis was conducted to explore differences between those who reported reasons versus those who did not. Among those who did not report a reason, there was a slightly higher proportion of people aged 46–54, who resided in an urban residence and had a mood disorder compared to those reporting a reason for not visiting an oral health professional (Table A1).

**Table 4:** Reasons for not visiting an oral health professional in the last 12 months stratified by dental insurance

	Overall n = 2,562		Insurance n = 842		No insurance n = 1,700	
	n	Weighted %	n	Weighted %	n	Weighted %
*Reasons for not visiting a dentist in the past 12 months* ^1^						
Not needed	2147	80.4%	668	76.2%	1462	82.5%
Difficulty getting an appointment	81	3.7%	35	5.5%	46	2.8%
No dentist, dental hygienst, denturist, or denturologist in the area	52	2.3%	24	2.6%	27	2.0%
Transportation problems	32	1.2%	11	1.2%	21	1.3%
Personal and family responsibilities	210	10.6%	86	11.9%	123	10.0%
Unable to leave the house due to health condition	40	1.9%	18	2.8%	21	1.4%
	Overall n = 43,959		Insurance* n = 26,885		No insurance* n = 16,862	
	n	Weighted %	n	Weighted %	n	Weighted %
*Did not go to dentist in past 12 months due to cost of care* ^2^						
Yes	4581	10.9%	1347	5.4%	3214	19.2%
No	39,378	89.1%	25,538	94.6%	13,648	80.8%

1. Participants could select all that apply

2. Participants were asked this question separately from other reasons for not visiting a dentist

*Significant difference between those who reported yes versus no (p < .0001)

Participants were also asked if the reason for not going to an oral health professional in the past 12 months was due to cost of care. Among people with no dental insurance, 19.2% reported this to be a reason for not visiting an oral health professional, compared to 5.4% of people who had dental insurance (Table 4).

## Discussion

This study identified several key factors that play a role in barriers to accessing oral health care, measured using last dental visit and dental insurance. For instance, people who reported not having any natural teeth were almost 10 times more likely to not have visited an oral health professional in the past 12 months, and 1.4 times more likely to not have dental insurance. Other factors suggested to be access barriers include age, household income, and rural residence. Although a small proportion of people who did not visit an oral health professional in the past 12 months reported the reason, most reported they did not need oral health care.

Similar to our study, an Ontario study found 76.0% of people aged 65 or older had visited an oral health professional in the past 12 months [18]. This study also noted that having dental insurance leads to a greater likelihood of visiting an oral health professional, thus leading to improved oral health [18]. The use of oral health care has been linked to a socioeconomic gradient, meaning people of lower income or lower education were less likely to visit an oral health professional [27, 28]. Given the consistency of findings related to barriers in accessing oral health care, it signifies the need to develop targeted preventive measures aimed at those who may face greater barriers.

It is apparent that dental insurance is a crucial component of access to oral health care [18]. The cost of treatment plays a crucial role in people receiving oral health treatment and care, as people often delay treatment due to the costs [29]. Income has been linked to access to dental insurance [30, 31], which is related to visiting an oral health professional [18]. This suggests the need for easier access to dental insurance plans, as access to insurance will increase oral health visits, thus improving oral health and the burden of disease associated with poor oral health.

Since only a few participants reported reasons for not visiting an oral health professional, it was difficult to draw conclusions. However, our findings indicate that most people did not visit an oral care professional as they felt they did not need to go. Research has found that people tend to perceive their oral health as good, but still have oral health problems [15]. It is important to develop population-based strategies aimed at educating people on the importance of regular oral care visits. This may include training health care professionals to better educate the importance of good oral health, and potential implications if oral health is not maintained [22]. In addition, a small proportion reported difficulties related to transportation as a reason for not visiting an oral health professional. This has been identified as a barrier to access in other studies [22], and governments may consider offering programs to enhance to ability to free transportation specifically for healthcare needs, such as oral health appointments.

The use of the CLSA to explore barriers to accessing oral health care is a strength, given the large sample size of aging Canadian adults. The CLSA contains rich data on oral health, as well as data on socioeconomic, psychosocial, and physical measures. Given the planned future waves of the CLSA, this study can be used to inform future research exploring the longitudinal impact of barriers to oral health. A limitation of this study was the cross-sectional design, making it difficult to infer temporality. Also, the characteristics of the CLSA, the large proportion of people who reported a high household income, or are highly educated, thus not making the sample representative of the broader Canadian population, however, findings from this study can be generalized to those who share similar characteristics to those in our study. The CLSA also does not include individuals living in institutions and in the Canadian territories, thus making it difficult to translate findings to these groups of people. A small proportion of people reported the reason for not visiting an oral health professional in the past 12 months was related to cost of care. This low proportion may be related to the participants included in the sample, as we may not have adequately included people of lower income who may have reported this as the reason for not receiving oral care. Another limitation includes the self-reported assessment of chronic conditions, anxiety, depression, and mood disorders. This could lead to misclassification of disease status potentially biasing results.

## Conclusions

To our knowledge, this is the first study to examine barriers accessing oral health care using a national population-based cohort of Canadian adults. Our study demonstrated that various factors impact duration since last oral health visit and dental insurance. Identifying these barriers is useful from a public health perspective when developing strategies to improve access to oral care, as targeted strategies can be created aimed at those not accessing oral health. For instance, the provision of access to dental insurance to high-risk groups is an example of an effort to increase access to oral health care which may contribute to a reduction of disease associated with poor oral health. Future research should consider exploring the mechanisms as to why these barriers lead to decreased access to oral care, as this can contribute to the development of targeted interventions further improving access to oral care.

## Electronic supplementary material

Below is the link to the electronic supplementary material.

Supplementary Material 1

## Data Availability

The data that support the findings of this study are available from the CLSA (https://www.clsa-elcv.ca/) but restrictions apply to the availability of these data, which were used under license for the current study, and so are not publicly available. Data are however available from the authors upon reasonable request and with permission of the CLSA.

## Declarations

### Disclaimer

The opinions expressed in this manuscript are the author’s own and do not reflect the views of the Canadian Longitudinal Study on Aging.

## List of Abbreviations

CI: Confidence Interval
CLSA: Canadian Longitudinal Study on Aging
OR: Odds Ratio
REB: Research Ethics Board
