# Supplementary material for: Barriers to oral care: a cross-sectional analysis of the Canadian longitudinal study on aging (CLSA)
Source: BMC Oral Health. 2023 May 15;23:294. doi: 10.1186/s12903-023-02967-3 (PMC10184348; doi:10.1186/s12903-023-02967-3)
Supplement: Supplementary file 1 — Supplementary Material 1 [file 12903_2023_2967_MOESM1_ESM.docx]

**APPENDIX**

**Table A1.** Sensitivity analysis comparing those who reported reasons for not visiting a dentist in the last 12 months versus those who did not report a reason^1^

| Characteristics | People who reported a reason for not visiting the dentist in the past 12 months  n=2,562 | | People who did not report a reason for not visiting the dentist in the past 12 months  n=3,950 | | p-value |
| --- | --- | --- | --- | --- | --- |
|  | n | Weighted % | n | Weighted % |  |
| Age group  46-54  55-64  65-74  75-92 | 222  630  752  958 | 12.4%  30.3%  30.1%  27.1% | 535  1111  1188  1116 | 21.3%  33.6%  26.0%  19.1% | 0.63 |
| Sex  Female  Male | 1211  1351 | 48.6%  51.4% | 1876  2074 | 47.7%  52.3% | <.0001 |
| Household Income  <$50,000  $50,000-$100,000  >$100,0000 | 1356  721  305 | 51.5%  33.4%  15.1% | 1911  1126  623 | 48.7%  32.8%  18.5% | <.0001 |
| Education  Less than post-secondary  Post-secondary | 1130  1414 | 43.2%  56.8% | 1475  2465 | 35.1%  64.9% | 0.06 |
| Residence  Rural  Urban | 565  1690 | 24.7%  75.3% | 314  3509 | 10.9%  89.1% | <.0001 |
| Smoking Status  None  Occasional  Daily | 2230  43  289 | 85.8%  1.6%  12.7% | 3365  73  507 | 88.4%  2.3%  14.4% | 0.16 |
| Mood Disorder  No  Yes | 2208  350 | 86.4%  13.6% | 3052  832 | 76.8%  23.3% | <.0001 |
| Anxiety  No  Yes | 2349  211 | 91.0%  9.0% | 3390  486 | 87.1%  12.9% | 0.0006 |
| Memory problem or dementia/Alzheimer’s  No  Yes | 2490  66 | 97.6%  2.4% | 3765  117 | 96.3%  3.7% | 0.05 |
| Chronic conditions  No  One or more | 191  2362 | 8.5%  91.5% | 260  3664 | 9.3%  90.7% | <.0001 |
| Social support  No  Yes | 525  1841 | 21.0%  79.0% | 1116  2700 | 27.3%  72.3% | 0.45 |
| Edentulous  No  Yes | 1525  1037 | 61.5%  38.5% | 2928  1021 | 80.8%  19.2% | <.0001 |
| Dental Insurance  No  Yes | 1700  842 | 66.6%  33.4% | 2536  1378 | 64.8%  35.2% | 0.31 |

1. Excluding the question asking about cost as a reason for not visiting the dentist
